# A Magnetic Resonance Imaging-Based Radiomic Model for the Noninvasive Preoperative Differentiation Between Transitional and Atypical Meningiomas

**DOI:** 10.3389/fonc.2022.811767

**Published:** 2022-01-21

**Authors:** Jing Zhang, Guojin Zhang, Yuntai Cao, Jialiang Ren, Zhiyong Zhao, Tao Han, Kuntao Chen, Junlin Zhou

**Affiliations:** ^1^ Department of Radiology, The Fifth Affiliated Hospital of Zunyi Medical University, Zhuhai, China; ^2^ Department of Radiology, Sichuan Provincial People’s Hospital, University of Electronic Science and Technology of China, Chengdu, China; ^3^ Department of Radiology, Affiliated Hospital of Qinghai University, Xining, China; ^4^ Department of Pharmaceuticals Diagnosis, GE Healthcare, Beijing, China; ^5^ Department of Radiology, Lanzhou University Second Hospital, Lanzhou, China; Key Laboratory of Medical Imaging of Gansu Province, Lanzhou, China

**Keywords:** meningioma, clinical decision-making, neoplasm grading, radiomics, retrospective studies

## Abstract

Preoperative distinction between transitional meningioma and atypical meningioma would aid the selection of appropriate surgical techniques, as well as the prognosis prediction. Here, we aimed to differentiate between these two tumors using radiomic signatures based on preoperative, contrast-enhanced T1-weighted and T2-weighted magnetic resonance imaging. A total of 141 transitional meningioma and 101 atypical meningioma cases between January 2014 and December 2018 with a histopathologically confirmed diagnosis were retrospectively reviewed. All patients underwent magnetic resonance imaging before surgery. For each patient, 1227 radiomic features were extracted from contrast-enhanced T1-weighted and T2-weighted images each. Least absolute shrinkage and selection operator regression analysis was performed to select the most informative features of different modalities. Subsequently, stepwise multivariate logistic regression was chosen to further select strongly correlated features and build classification models that can distinguish transitional from atypical meningioma. The diagnostic abilities were evaluated by receiver operating characteristic analysis. Furthermore, a nomogram was built by incorporating clinical characteristics, radiological features, and radiomic signatures, and decision curve analysis was used to validate the clinical usefulness of the nomogram. Sex, tumor shape, brain invasion, and four radiomic features differed significantly between transitional meningioma and atypical meningioma. The clinicoradiomic model derived by fusing the above features resulted in the best discrimination ability, with areas under the curves of 0.809 (95% confidence interval, 0.743-0.874) and 0.795 (95% confidence interval, 0.692-0.899) and sensitivity values of 74.0% and 71.4% in the training and validation cohorts, respectively. The clinicoradiomic model demonstrated good performance for the differentiation between transitional and atypical meningioma. It is a quantitative tool that can potentially aid the selection of surgical techniques and the prognosis prediction and can thus be applied in patients with these two meningioma subtypes.

## Introduction

Meningiomas are the most common primary intracranial tumors in adults, accounting for 36.7% of all intracranial tumors (1). According to the latest 2016 edition of the World Health Organization (WHO) classification of central nervous system tumors (2), meningiomas have been classified into 3 grades and 15 different subtypes. Among these different subtypes, transitional meningioma (TM) is a common benign meningioma (WHO grade I), whereas atypical meningioma (AM) is an uncommon tumor of intermediate grade between benign and malignant forms (WHO grade II). Pathologically, TM is characterized by the transitional morphological manifestation between endothelial meningiomas and fibrous meningiomas (3). AM is defined as a tumor with increased mitotic activity (≥4 mitoses per 10 high-power fields), brain invasion, and at least three of the following minor criteria: increased cellularity, high nucleus-to-cytoplasm ratio, prominent nucleoli, sheet-like architecture, and spontaneous necrosis foci (4, 5).

According to the European Association of Neuro-Oncology (EANO) guidelines, magnetic resonance imaging (MRI) is the main method used in the provisional diagnosis of meningiomas (6). At present, several studies have explored imaging features to assess the tumor grade, and some imaging features (such as tumor heterogeneity, shape, and tumor-brain interface) may be used as predictive factors to discriminate between tumors of different grades (7–9). Zhang et al. used MRI features to distinguish some subtypes of WHO grade I meningiomas (angiomatous, meningothelial, fibroblastic, and psammomatous meningiomas) and found that angiomatous and meningothelial meningiomas were the most easily identifiable subtypes (10). However, current image-guided evaluation depends on the experience of radiologists, which is non-specific and highly subjective. Recently, our previous study (11) has shown that among meningiomas, WHO grade I TM and WHO grade II AM are more aggressive than other subtypes because the frequency of brain invasion in these two tumors was much higher than in other subtypes. This study showed that TM was more aggressive than other subtypes of WHO grade 1 meningioma, and its biological behavior is close to that of atypical meningioma. Another study observed that several imaging characteristics, such as irregular tumor shape, heterogeneous contrast enhancement, and peritumoral edema were identified as predictors of brain invasion (12). The above research suggests that TM and AM may be similar in their imaging presentation, although the reported data on TM remain scarce, especially regarding its imaging characteristics. However, the clinical treatment plan and prognosis of these two tumors are significantly different due to their different grades. According to EANO guidelines for the treatment of meningiomas, the diagnosis of WHO grade II meningioma (such as AM) implies an increased risk of recurrence, requiring shorter control intervals (every 6 months instead of annually) than in WHO grade I TM (6). Han et al. reported that TMs can be treated with either surgery or external beam radiation, AMs often require a combination of the two modalities (13). The choice of surgical technique may be different. Because AM is more prone to invasive growth and recurrence. Whether to expand the scope of surgical resection, application of intraoperative navigation and preoperative blood preparation, this is closely related to the size of the tumor (AM tends to be slightly larger than TM) and tumor surrounding tissues Moreover, it has been established that higher tumor grades are associated with worse prognosis; higher grades indicate reduced survival and higher rates of tumor recurrence (14). Therefore, precise distinction between TM and AM before surgery is desirable.

Given the above reasons, it is necessary to explore the imaging differences between AM and TM. Radiomic analysis is a reliable tool that can quantify high-dimensional tumor features that cannot be observed with the naked eye, such as intensity, texture, and shape features (15, 16). In recent years, radiomic analysis has rapidly transformed the field of medical imaging analysis, since it provides more stable results and is an objective rather than a subjective assessment. Several studies have demonstrated the applications of radiomics in meningiomas, such as the characterization of the grade and histological subtype, the prediction of brain invasion and recurrence-free survival, and the identification of differential diagnoses in meningioma (11, 17–20). These studies show that the MRI-based radiomics may also be a method for discriminations between AM and TM.

To the best of our knowledge, this is the first study to differentiate TM from AM based on texture feature or radiomic analysis. Therefore, our study aimed first to identify MR and radiomic features that are associated with these two tumors from two MRI modalities [T2-weighted (T2) and T1-weighted post-contrast (T1C)]; second, to combine these two modalities generating a radiomic signature; and third, to build a nomogram fusing clinical factors, MR features, and radiomic signatures to differentiate TM from AM in MRIs of patients with suspected meningioma.

## Materials and Methods

### Study Population and Semantic Features

For this retrospective analysis, ethical approval was obtained from the Institutional Review Board of Lanzhou University Second Hospital, and the requirement for informed consent was waived. In this study, all patients with TM and AM who underwent surgery in our institute between January 2015 and December 2019 were enrolled according to the following inclusion and exclusion criterias. The inclusion criteria were: (a) histological diagnosis of AM or TM, and (b) MRI, including T1C and T2 sequences, performed within 1 week before surgical tumor resection. The exclusion criteria were: (a) cases with motion artefacts that impacted the assessment; (b) incomplete MRI sequences; and (c) treatment such as radiotherapy, chemoradiotherapy, or surgery before surgical tumor resection.

**Figure 1:**
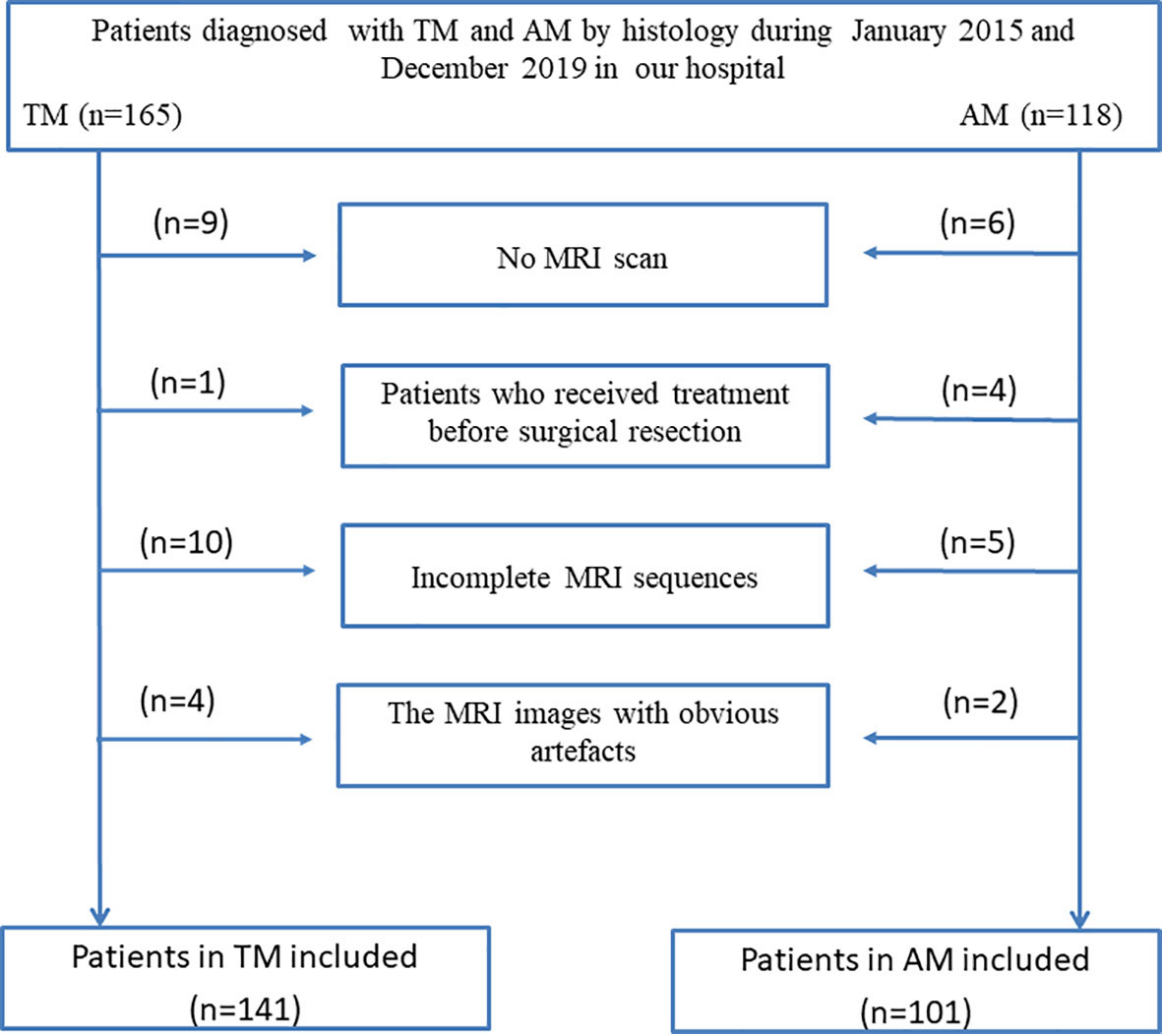
Inclusion and exclusion criteria.

All tumors were resected with the aid of a microscope. Patients with TM and AM were diagnosed according to the pathological findings. Finally, a total of 242 patients (TM: 19 men, 122 women, mean age 52.3 ± 9.2 years; AM: 46 men, 55 women, mean age 51.5 ± 10.3 years) were enrolled. All patients were randomly divided into a training cohort and a validation cohort in a 7:3 ratio. The patient recruitment flowchart is shown in **Figure 1**.

Two radiologists (reader 1 JZ and reader 2 YTC, with 12 and 15 years of experience in brain MRI interpretation, respectively) independently analyzed the MRI characteristics (including tumor location, maximum diameter, tumor shape, tumor border, dural tail sign, peritumoral edema, T2 signal, enhanced features, bone invasion, sinus invasion, and brain invasion). The image analysis was based on clinical experience. Both readers were blinded to all personal information and the histopathological results before analysis. For qualitative data, agreements were reached after discussion between the two in cases of difference of opinions. When the two readers were unsure, reader 3 (ZYZ) with 19 years of experience confirmed the results. For quantitative data, reader 1 measured the maximum diameter three times on the maximum level of the tumor, and calculated the average of three measurements. Reader 2 performed the data measurement in the same way. The final result was the average of the measurement values of two readers to minimize the deviation of the measurement results. Among MR features evaluation, peritumoral edema was evaluated on T2 images according to the standardized visually accessible Rembrandt Images (VASARI; https://wiki.nci.nih.gov/display/CIP/VASARI) feature set. Brain invasion was diagnosed by pathology. Bone invasion assessments were performed by pathology and surgeon assessment intraoperatively. Sinus invasion was evaluated by an intraoperative neurosurgeon as a diagnostic standard.

### Image Acquisition, Segmentation, and Normalization

The MRIs were obtained at our institution with 3.0-T scanners (Siemens Verio or Philips Achieva). The MR sequences included T1C and T2 images, and the detailed parameters of each scanner are shown in **Supplementary Table S1**.

Two radiologists (TH and JZ) without prior knowledge of the pathological records manually segmented MR images using the open-source software ITK-SNAP (version: 3.8.0, www.itksnap.org). On both axial T1C and T2 images, the regions of interest (ROIs) of images were manually delineated on each slice of the entire tumor including hemorrhagic, necrotic lesions and without the surrounding brain tissue, and oedema. T2 images were segmented with reference to T1C images for visual guidance. The segmented tissues on each slice were fused together to generate the volume of interest, as shown in **Supplementary Figure 1**.

To obtain a standard normal distribution of the image intensities, T1C and T2 images were standardized using z-score normalization and resampling after manual segmentation. MR scanners and image segmentation of two additional sets (i.e. two MR scanners set and the re-segmentation set) are described in Supplementary Material.

### Feature Extraction and Selection

The PyRadiomics platform was used to extract standardized radiomic features from the T1C and T2 imaging data (21). In this study, feature extraction followed the Image Biomarker Standardization Initiative (IBSI) guideline (22). T1C features were extracted from the volume of interest (VOI) of T1C images, whereas T2 features were extracted from the VOI of T2 images. Finally, a total of 2454 radiomic features were extracted from the VOI of two modalities of the MR images.

For both T1C and T2 features, the least absolute shrinkage and selection operator (LASSO) regression with five-fold cross-validation was conducted to select the radiomic features highly correlated with discrimination of TM and AM (**Supplementary Figure 2**). Features with a *P-*value of less than 0.05 were selected. For clinical factors and MRI features, the correlation between these two factors and discrimination of AM and TM were tested *via* Student’s t-test and the chi-square test with the *P*-value set to 0.05. Then, stepwise multivariate logistic regression further selected the most informative features and deleted irrelevant features. Features with a *P*-value of less than 0.05 and preoperative factors were included in the model. Spearman correlation analysis was conducted to examine the correlation between the selected radiomic features and clinicoradiological features to determine whether these features are correlated with each other.

### Fusion of Modalities and Radiomic Signature Building

T1C represents the blood supply and the integrity of the blood-brain barrier, whereas T2 is sensitive to peritumoral edema, thus mainly reflecting tissue edema. Therefore, these two modalities were fused by combining the selected radiomic features to increase the performance of the radiomic model. After fusing the modalities, we used stepwise multivariate logistic regression to build a radiomic model discrimination of TM and AM based on the selected radiomic features. The T1C model was built based on T1C features (two features), and the T2 model was built based on T2 features (two features), whereas the fusion model was built based on T1C and T2 fusion features (all four radiomic features). The clinical model was built based on a clinical factor (sex) and MRI features (tumor shape and brain invasion). Thus, the clinicoradiomic model was built by incorporating the clinical factor, MRI features, and the radiomic signature. In the training cohort, the maximum area under the receiver operating characteristic curve (AUC) with three-fold cross-validation determined the final regularization parameter.

### Nomogram Building and Validation

Integrated discrimination improvement (IDI) (23) was used to quantify performance improvements. The *P*-values indicated whether the improvement in reclassification was statistically significant after the inclusion of a new factor in the model. In addition, we used the DeLong test to compare the AUC estimates of the performance between different models.

Afterward, a nomogram for clinical usefulness incorporating the radiomic signature and the correlated clinicoradiological features was constructed in the training cohort and validated in the validation cohort. The calibration curves assessed the discrimination ability of the nomogram for the training and test cohorts, and the Hosmer-Lemeshow test evaluated the agreement between the discrimination of TM from AM and the observed outcomes. Then, we used decision curve analysis (DCA) to quantify the net benefits at different threshold probabilities to evaluate the clinical efficacy of the nomogram (24).

### Statistical Analysis

In this study, all statistical analyses were performed with R software (version 3.6.4, http://www.Rproject.org). R was also used to assess the prediction models. PyRadiomics was used to extract and select the radiomic features, as well as to build the prediction models. The Spearman correlation test was used to explore differences between clinicoradiological features and radiomic features. Student’s t-test and the chi-square test were used to compare continuous and categorical variables, respectively. Generally, two-sided *P*-values less than 0.05 were considered statistically significant. The intra-/inter-class correlation coefficients (ICCs) were used to assess the agreement of the two MR scanners and the extracted features by two radiologists.

## Results

### Clinical Factors and MR Features

The clinical factors and MR features of the patients are shown in **Table 1**. For clinical factors, sex was found to be significantly different (*P* < 0.001) between the TM and AM groups, whereas age did not differ significantly (*P* > 0.05). For MR features, the parameters maximum diameter, tumor shape, peritumoral edema, enhanced features, bone invasion, and brain invasion were significantly different (all *P* < 0.05) in the univariate analysis. Among them, tumor shape and brain invasion were highly correlated with discrimination of TM from AM and can be used as independent predictive factors according to the multivariate logistic regression analysis. By contrast, tumor location, tumor border, dural tail sign, T2 signal, and sinus invasion were not significantly different (all *P* > 0.05) between the TM and AM groups.

**Table 1:** Clinical factors of the patients and magnetic resonance imaging features in the training and validation cohorts.

Characteristics	AM (n = 101)	TM (n = 141)	Univariate analysis (*p* value)	Multivariate analysis (*p* value)
Clinical factors				
Age (years)	51.5 ± 10.3	52.3 ± 9.2	0.543	N/A
Sex			<0.001*	<0.001*
Female	55 (54.5%)	122 (86.5%)		
Male	46 (45.5%)	19 (13.5%)		
Imaging features				
Tumor location			0.442	N/A
Parasinus and parasial	56 (55.4%)	69 (48.9%)		
Skull base	28 (27.8%)	50 (35.5%)		
Convexity	17 (16.8%)	22 (15.6%)		
Maximum diameter (mm)	47.96 ± 15.89	37.36 ± 15.18	<0.001^*^	N/A
Tumour shape			<0.001^*^	<0.001*
Circular or quasi- circular	38 (37.6%)	89 (63.1%)		
Irregular	63 (62.4%)	52 (36.9%)		
Tumour border			0.106	N/A
Clear	81 (80.2%)	124 (87.9%)		
Blur	20 (19.8%)	17 (12.1%)		
Dural tail sign			0.175	N/A
Yes	41 (40.6%)	45 (31.9%)		
None	60 (59.4%)	96 (68.1%)		
Peritumoural oedema			<0.001^*^	N/A
None (0%)	27 (27.3%)	83 (58.9%)		
≤5%	23 (23.2%)	19 (13.5%)		
6-33%	19 (19.2%)	14 (9.9%)		
34-67%	17 (17.2%)	19 (13.5%)		
68-95%	13 (13.1%)	6 (4.3%)		
MRI signal				
T2WI			0.056	N/A
Slightly high signal	50 (49.5%)	59 (41.8%)		
Iso signal	31 (30.7%)	64 (45.4%)		
Mixed signal	20 (19.8%)	18 (12.8%)		
Enhanced features			<0.001^*^	N/A
Uniform	42 (41.6%)	93 (66.0%)		
Uneven enhancement	59 (58.4%)	48 (34.0%)		
Bone invasion			0.022^*^	N/A
Yes	38 (37.6%)	33 (23.4%)		
No	63 (62.4%)	108 (76.6%)		
Sinus invasion			0.469	N/A
Yes	25 (24.8%)	41 (29.1%)		
No	76 (75.2%)	100 (70.9%)		
Brain invasion			0.014*	0.011*
Yes	21 (20.8%)	13 (9.2%)		
No	80 (79.2%)	128 (90.8%)		

Among peritumoural oedema, percentage represents the proportion of peritumoural oedema in the entire abnormality, and the entire abnormality may be comprised of the entire tumour and oedema component.T2 signal is defined by comparing the signal of the gray matter of the brain. A Student’s t-test was used to compare the difference in age and maximum diameter, while the chi-square test was used to compare the difference in other features. *P < 0.05. SD, standard deviation. N/A, not available.

### Radiomic Features Correlated With TM and AM

**Figure 2:**
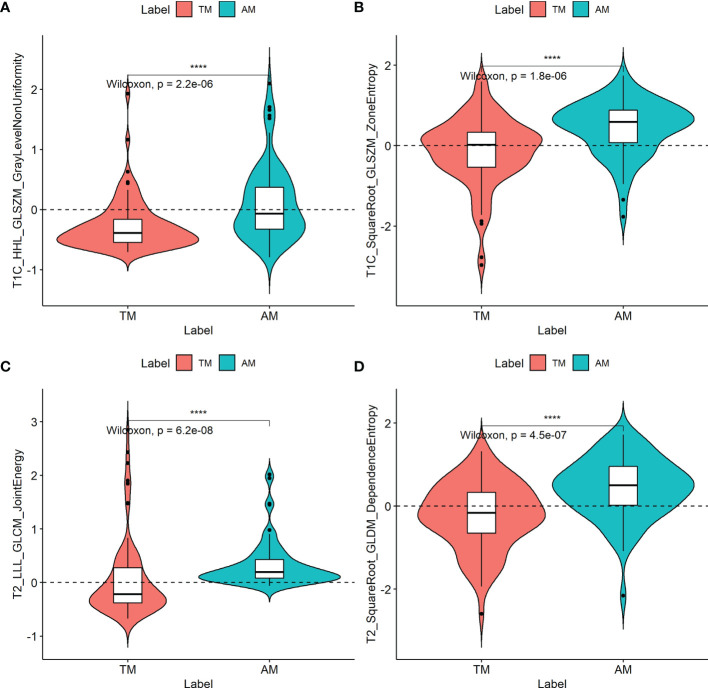
Boxplots of the four radiomic features **(A–D)** with significant differences between transitional meningioma (TM) and atypical meningioma (AM) groups in the training cohort. The symbol **** represents *p* < 0.001.

The ICCs were calculated to evaluate the agreement of the two MR scanners and the features extracted by two radiologists, respectively. All values exceeded 0.75, reflecting good agreement. In total, 2454 radiomic features were extracted from each patient. Among them, two T1C features and two T2 features were selected, and all four radiomic features (T1C_WaveletGLSZMwavelet.HHL_GraylevelNonUniformity, T1C_SquareRootGLSZM_squareroot_zoneEntropy, T2_WaveletGLCMwavelet.LLL_JointEnergy, and T2_SquareRootGLDM_squareroot_DependenceEntropy) were significantly different between the TM and AM groups (all *P* < 0.05; **Figure 2**). Their odds ratios are shown in **Supplementary Figure 3**. The weights of each selected radiomics features are shown in **Supplementary Table 2**.

**Figure 3:**
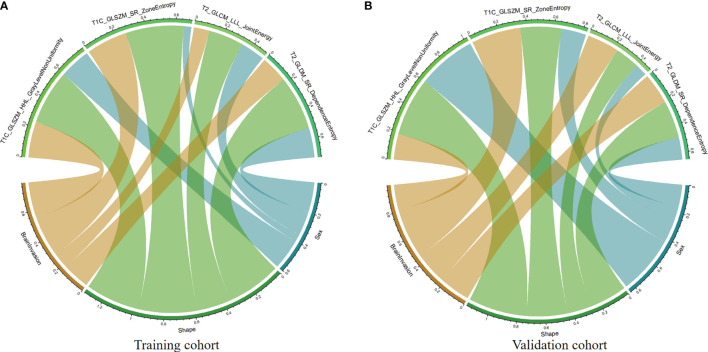
Chord diagram of the correlation between clinicoradiological and selected radiomic features. Correlation analysis of clinicoradiological and selected radiomic features in the training **(A)** and validation **(B)** cohorts. The Spearman correlation test confirms that each link is significantly correlated (*P* < 0.05). The width of a link represents the strength of the correlation. For example, the T1C_SquareRootGLSZM_squareroot_zoneEntropy feature (gray) is highly correlated with tumor shape in both training and validation cohorts.

According to the Spearman correlation test, these four features extracted by algorithms from MR images were consistent with some clinicoradiological features evaluated by the radiologists (**Supplementary Table 3**). For example, shape was correlated with the parameters T1C_WaveletGLSZMwavelet.HHL_GraylevelNonUniformity and T1C_SquareRootGLSZM_squareroot_zoneEntropy in both training and validation cohorts (**Figure 3**).

### Fusion of Modalities and Model Building

**Figure 4:**
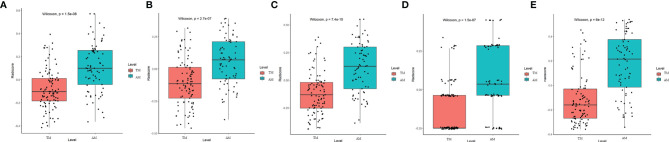
Plots **(A–E)** show the boxplots of the corresponding radiomics score in the T1C, T2, combination of T1C and T2, clinical and clinicoradiomics models, respectively.

T1C and T2 radiomic features may correspond to different information. Though fusing the selected radiomic features, the radiomics signature can reflect the discrimination factors of TM and AM from different perspectives. Stepwise multivariate logistic regression analysis showed that sex, tumor shape, and brain invasion were significantly different between TM and AM groups (all *P* < 0.001). Thus, the radiomic signature, sex, tumor shape, and brain invasion were selected for the clinicoradiomic model building. The radiomics scores in the AM group were significantly higher than those in the TM group in the different models, as shown in the **Figure 4**.

**Figure 5:**
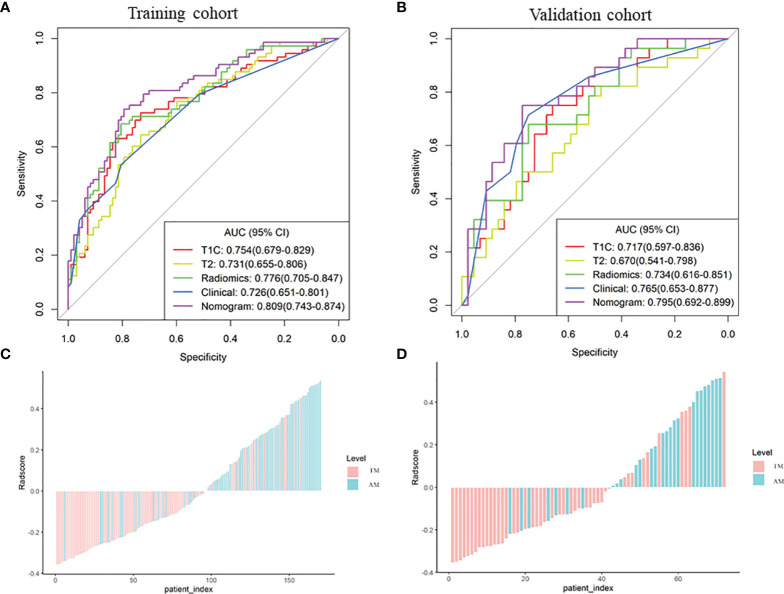
Comparison of the receiver operating characteristic (ROC) curves of the different models. **(A, B)** ROC curves of the different models in the training and validation cohorts. The clinicoradiomic model demonstrates the best discrimination ability among these models, with area under the curve (AUC) values of 0.809 and 0.795 in the training and validation cohorts, respectively. **(C, D)** Radiomic signature histogram of the training and validation cohorts. The red bar shows the sample with transitional meningioma (TM), and the blue bar shows the sample with atypical meningioma (AM).

The performance of these models was evaluated in the training cohort and then validated in the validation cohort. The discrimination ability of T1C, T2, the radiomic (fusion of T1C and T2), clinical, and the clinicoradiomic models are shown in **Table 2**. The ROC curves for T1C, T2, the radiomic, clinical, and clinicoradiomic models are plotted in **Figure 5**. The clinicoradiomic model (nomogram) demonstrated the best discrimination ability, resulting in AUCs of 0.809 (95% CI, 0.743-0.874) and 0.795 (95% CI, 0.692-0.899) with sensitivity values of 74.0% (95% CI, 49.3%-83.6%) and 71.4% (95% CI, 42.9%-89.3%) for the differentiation of TM from AM in the training and validation cohorts, respectively. The formula for calculating the clinicoradiomic model and the fusion radiomic signature is described respectively in the **Supplementary Results**.

**Table 2:** Performance of the sequence models.

Cohort	Model	AUC	ACC	SEN	SPE	PPV	NPV
Training set	T1C	0.754 (0.679-0.829)	0.729 (0.656-0.795)	0.726 (0.589-0.822)	0.732 (0.464-0.866)	0.671 (0.623-0.698)	0.780 (0.692-0.808)
	T2	0.731 (0.655-0.806)	0.694 (0.619-0.762)	0.644 (0.466-0.767)	0.732 (0.556-0.825)	0.644 (0.567-0.683)	0.732 (0.675-0.755)
	Radiomics	0.776 (0.705-0.847)	0.753 (0.681-0.816)	0.685 (0.507-0.795)	0.804 (0.464-0.887)	0.725 (0.661-0.753)	0.772 (0.662-0.789)
	Clinical	0.726 (0.651-0.801)	0.688 (0.613-0.757)	0.534 (0.380-0.639)	0.804 (0.685-0.900)	0.672 (0.593-0.711)	0.696 (0.661-0.720)
	Nomogram	0.809 (0.743-0.874)	0.771 (0.700-0.831)	0.740 (0.493-0.836)	0.794 (0.567-0.866)	0.730 (0.643-0.753)	0.802 (0.743-0.816)
Test set	T1C	0.717 (0.597-0.836)	0.694 (0.575-0.798)	0.750 (0.392-0.893)	0.659 (0.295-0.818)	0.583 (0.423-0.625)	0.806 (0.650-0.837)
	T2	0.670 (0.541-0.798)	0.611 (0.489-0.724)	0.607 (0.392-0.858)	0.614 (0.432-0.886)	0.500 (0.392-0.586)	0.711 (0.633-0.780)
	Radiomics	0.734 (0.616-0.851)	0.722 (0.604-0.821)	0.679 (0.285-0.857)	0.750 (0.431-0.864)	0.633 (0.420-0.686)	0.786 (0.678-0.809)
	Clinical	0.765 (0.653-0.877)	0.736 (0.619-0.833)	0.714 (0.372-0.879)	0.750 (0.499-0.864)	0.645 (0.486-0.691)	0.805 (0.733-0.826)
	Nomogram	0.795 (0.692-0.899)	0.750 (0.634-0.845)	0.714 (0.429-0.893)	0.773 (0.477-0.910)	0.667 (0.545-0.714)	0.810 (0.724-0.833)

T1C, contrast-enhanced T1-weighted imaging; T2WI, T2-weighted imaging; Radiomics, combination of T1C and T2; Clinical, fusion of sex, tumour shape and brain invasion; AUC, area under receiver operating characteristic curve; ACC, balanced accuracy; SEN, sensitivity; SPE, specificity; PPV, positive predictive value; NPV, negative predictive value.

### Model Comparison

The IDI index was calculated to assess the predictive usefulness of the different models. The clinicoradiomic model improved the integrated discrimination by 5.75% (*P* = 0.002) and 9.96% (*P* < 0.001) compared to the radiomic model in the training and validation cohorts, respectively. The comparisons between different models are shown in **Table 3**. In addition, Delong test showed that compared with Clinical and T2 models, the discrimination ability of clinicoradiomic model has been significantly improved in the training cohort, *P* value was 0.014 and 0.004 respectively, and there is no statistical significance in the validation cohort.

**Table 3:** Comparison of the different models in the validation cohort.

Initial model	Model introducing new factor	Performance improvement (IDI)	
		Training cohort	Validation cohort
Clinical	Clinicoradiomic	11.1% *P* = 0.00033	7.86% *P* = 0.0396
Combination of T1C and T2	Clinicoradiomic	5.75% *P* = 0.00195	9.96% *P* = 0.00089
T1C	Combination of T1C and T2	4.37% *P* = 0.00538	3.06% *P* = 0.3425
T2	Combination of T1C and T2	7.04% *P* = 0.00029	6.91% *P* = 0.02442

Compared with the T1C and T2 models, the performance of combination of T1C and T2 model improved by 7.04% and 4.37% in discrimination ability, respectively. Compared with combination of T1C and T2 model, the performance of clinicoradiomic model improved by 5.75% in discrimination ability. IDI: Integrated discrimination improvement; Clinicoradiomic, fusion of sex information, tumour shape, brain invasion and radiomic signature.

### Assessment of the Clinicoradiomic Nomogram Performance

**Figure 6:**
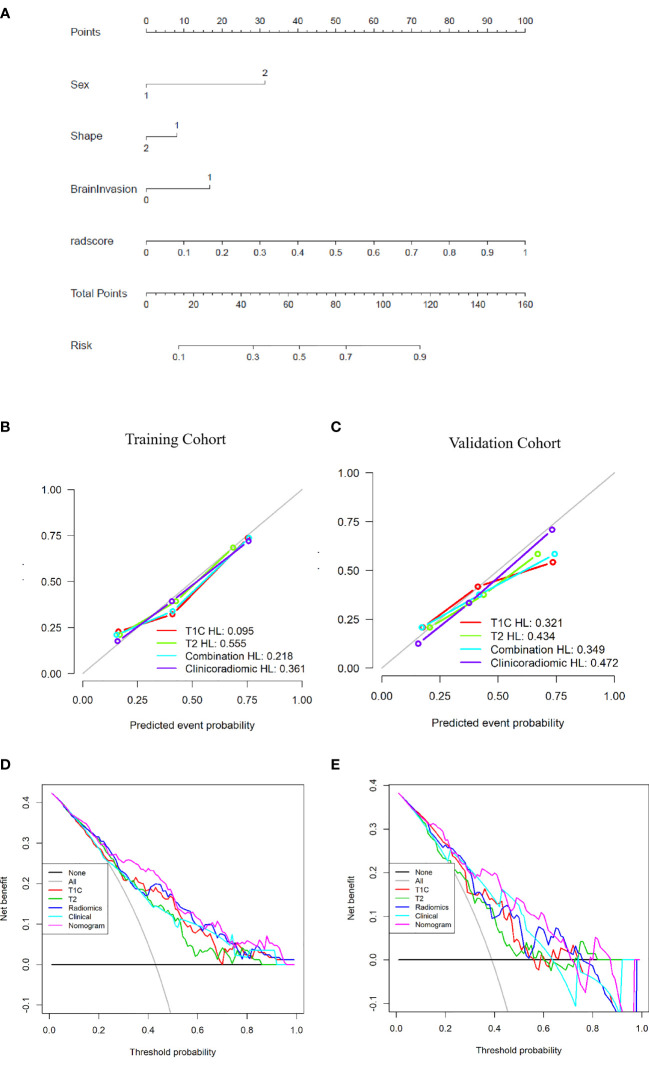
Establishment and performance of the clinicoradiomic model. **(A)** The clinicoradiomic model is used to develop a nomogram. **(B, C)** Calibration curves of the clinicoradiomic nomogram for the training and validation cohorts. The x-axis represents the probability of atypical meningioma (AM) and transitional meningioma (TM) as measured by the clinicoradiomic model, and the y-axis represents the actual rate of AM and TM. The solid line represents the discrimination ability of the nomogram, and the diagonal dotted line represents the ideal evaluation by a perfect model. The *P*-values in the Hosmer-Lemeshow test are 0.361 and 0.472 in the training and validation cohorts, respectively. A closer fit to the diagonal dotted line represents a better evaluation. **(D, E)** Decision curve analysis for the clinicoradiomic model. The x-axis shows the threshold probability, and the y-axis measures the net benefit. The gray line represents all patients with AM, whereas the black line represents all patients with TM. The pink line represents the clinicoradiomic model.

The clinicoradiomic model demonstrated the best discrimination ability and was used to construct the nomogram (**Figure 6A**). The calibration curve together with the Hosmer-Lemeshow test were used to measure the consistency between the probability of TM or AM being diagnosed by the clinicoradiomic model and the actual pathological diagnosis. The actual pathological diagnosis was consistent with the predicted probability of TM and AM in both the training and validation cohorts, with *P*-values of 0.361 and 0.472, respectively, as shown in **Figures 6B, C**.

The DCA assessed the discrimination ability of the clinicoradiomic model based on clinical applications. The clinicoradiomic model provided a net benefit in the DCA at a threshold probability of above 20% (**Figures 6D, E**). This result indicated that the clinicoradiomic data were clinically useful.

## Discussion

This is a preliminary study to develop a clinicoradiomic model that discriminates TM from AM based on MRI. The discrimination ability of this fusion model was validated *via* DCA, discrimination, and calibration curves in an internal validation cohort. One clinical factor, two radiological features, and four radiomic features indicated a high correlation with the ability of a model to discriminate between TM and AM. A multi- modality (fusion of T1C and T2) model of radiomics showed good discrimination ability in both training (AUC: 0.776, Sensitivity: 0.685) and validation (AUC: 0.734, Sensitivity: 0.679) cohorts. Moreover, the nomogram incorporating clinicoradiological and radiomic features demonstrated the best performance in both training (AUC: 0.809, Sensitivity: 0.740) and validation (AUC: 0.795, Sensitivity: 0.714) cohorts.

Among clinical factors, sex was only the parameter that was significantly different between TM and AM. Females (86.5%) were prone to TM, whereas the male-to-female ratio was balanced in AM (females 54.5%), which is consistent with the results of other studies (3, 25). Among MR features, tumor shape and brain invasion were significantly different between TM and AM, and based on the stepwise multivariate logistic regression analysis, they can be used as independent discrimination factors. AMs are more irregular, and TMs are mostly circular or quasi-circular, which may be related to the grade of the tumor and the increased brain volume due to the peritumoral edema (26). Similarly, in most studies, irregular or lobulated tumor growth was associated with high-grade histology in both uni- and multivariate analyses (7, 8, 27, 28), presumably showing a parenchymal reaction of the brain tissue to the extensive tumors growth and the aggressiveness of the meningioma (27). Some authors also found that irregular or lobulated meningiomas were more likely to recur than regular-shaped ones (7). Zhang et al. have reported that the frequencies of brain invasion in TM and AM were much higher than those in other meningioma subtypes. In our study, the incidence of brain invasion in AM (20.8%) was higher than that in TM (9.2%), which is consistent with a previous study (4%-19% in all WHO grade meningiomas) (11, 12, 29). This indicates that WHO grade II AMs are more aggressive than WHO grade I TMs. Moreover, the maximum diameter, peritumoral brain edema, heterogeneous enhancement, and bone invasion were also different in these two tumors according to the univariate analysis, in agreement with published reports (30). For example, larger tumor size and tumor volume were more likely to be observed in high-grade meningiomas, and AM is a WHO grade II tumor. Heterogeneous enhancement reflects intratumoral hemorrhage, ischemic necrosis, cystic change, or calcification and is associated with heterogeneous distribution of tumor cells. Previous studies have reported that AMs have significantly more intratumoral necrosis and cystic changes than benign meningiomas (1, 31).

At present, MR radiomics can reproducibly extract objective and quantitative data from different sequences (T2, T1, T1C, and fluid attenuated inversion recovery [FLAIR], among others) to diagnostically discriminate meningiomas from other tumor forms, such as craniopharyngioma from meningioma in the sellar/parasellar area (32) or malignant hemangiopericytoma from angiomatous meningioma (20, 33). Radiomics can use visually imperceptible information about the tumor. Given this background, the radiomics model is a convenient, noninvasive method that does not require tissue biopsy or gene sequencing and may be a valuable approach to differentiate TM from AM since the radiomics model (AUC: 0.776 in the training cohort) outperformed the clinical model (AUC: 0.726). Additionally, we developed and validated a clinicoradiomics model to discriminate between TM and AM. Of the 2454 radiomic features, four were highly correlated with the discrimination between these two tumors. These features were textural image features indicating microscopic descriptions of the tumor including cellularity and tumor-induced compression of normal brain tissue. Textural features can neither be identified by the human visual system nor be easily interpreted to understand their specific meaning (7, 34, 35). We analyzed the four identified radiomic features and found that two gray-level size zone matrix (GLSZM) features, one gray-level dependence matrix (GLDM) feature, and one gray-level co-occurrence matrix (GLCM) feature were significantly associated with the discrimination of TM from AM. According to the definitions of these texture features (36), GLSZM quantifies gray-level zones in an image. The GLDM feature measures the difference between adjacent voxels based on their voxel value, and this feature was most relevant to the discrimination between these two tumors. The GLDM features selected by LASSO include entropy features, where a larger entropy value indicates greater heterogeneity of the tumor (37). The GLCM feature describes the distance and angle of each pixel, which includes energy, correlation, entropy, inertia, and inverse difference (37). Compared to TM, the values of these features were higher in AM. This indicates that these features may reflect microscopic heterogeneity within the tumors. Thus, as a new tool, the radiomic feature could distinguish TM from AM.

We also analyzed the correlation between clinicoradiological factors and radiomic features using Spearman’s correlation analysis. We found that some clinicoradiological factors were consistent with some radiomic features extracted from MR images in both training and validation cohorts. This revealed that clinicoradiological features also reflected some radiomic MRI features. For example, shape was correlated with T1C_WaveletGLSZMwavelet.HHL_GraylevelNonUniformity and T1C_SquareRootGLSZM_squareroot_zoneEntropy in both training and validation cohorts. This indicated that some radiomic features corresponded to tumor shape in MR images, such as entropy in GLSZM. The correlation between these features revealed that some specific feature combinations may be explained by some clinicoradiological features to some extent. Besides, the included four features were extracted from different sequences, and the combination of T1C and T2 models of radiomic features indicated a better discrimination ability than the T1C or T2 models alone. These results indicated that different sequences provided distinct information, and multiple sequences could show more information about tumors and increase the discrimination ability of the model. After Delong test, we found that the discrimination ability of clinicoradiomic model is better than that of Clinical and T2 models. However, there is no statistical significance in the validation cohort, which may be related to the small sample size of the validation cohort. This result indicated that we should include more data in future study to improve the performance and stability of the model. At present, the clinical application of radiomics is still in the exploratory research stage, and it needs time to accumulate. Radiomics-derived data, when combined with other pertinent data sources (including clinically obtained, treatment-related or genomic data), can produce accurate robust evidence-based clinical-decision support systems (38).

The nomogram incorporating clinicoradiological factors and radiomic features showed the best discrimination ability compared to radiomic models based on T1C, T2, T1C/T2 images, and clinicoradiological factors. The results indicated that fusing clinicoradiological factors and radiomic features significantly improved classification performance. Combining qualitative and quantitative imaging analyses provided an additive effect because the information contained in these features was complementary (39). Preoperative risk factors were extracted without postoperative factors to build the nomogram. This nomogram may be helpful for both clinicians and radiologists to preoperatively distinguish TM from AM. The nomogram was better than the radiomics model and could be applied in clinical practice for meningioma patients undergoing MRI scans.

Our study has several limitations. First, the analysis of MRI characteristics was independently performed by two radiologists, and imaging assessments are subjective. Second, we selected the T1C and T2 sequences for this study. On T2 images, the tumor had unclear boundaries in some cases. Although we referred to the T1C sequences for visual guidance to delineate tumor borders, there were still deviations. Third, our study was a single-center study, and a multi-center external validation is needed to test the generalizability and robustness of the model in the future. In addition, two neuroradiologists spent plenty of time to manually delineate two independent VOIs of tumors for each MRI sequence in this study, thus, efficient automatic segmentation and co-registration was available for meningiomas in the future research. In future, multimodal studies such as DWI and FLAIR sequences could be combined to improve accuracy.

## Conclusion

Preoperative identification of TM and AM would aid the clinical decision-making and prognosis prediction. In the radiomic analysis, four radiomic features were highly correlated with the discrimination between these two tumors. After the fusion of T1C and T2 features, the MRI-based radiomics signature effectively identified TM and AM on MR images. The clinicoradiomic model that combined the radiomic signatures and clinicoradiological factors showed the best discrimination ability and may be used in patients with TM and AM.

## Data Availability Statement

The original contributions presented in the study are included in the article/**Supplementary Material**. Further inquiries can be directed to the corresponding authors.

## Ethics Statement

The studies involving human participants were reviewed and approved by For this retrospective analysis, ethical approval was obtained from the Institutional Review Board of Lanzhou University Second Hospital, and the requirement for informed consent was waived. Written informed consent for participation was not required for this study in accordance with the national legislation and the institutional requirements.

## Author Contributions

JZ, GZ, JLZ, KC, and YC contributed to conception and design of the study. TH organized the database. JR performed the statistical analysis. JZ wrote the first draft of the manuscript. ZZ, JR, YC, and GZ wrote sections of the manuscript. All authors contributed to manuscript revision, read, and approved the submitted version.

## Funding

This study was supported by grants of National Natural Science Foundation of China (82071872), Doctoral Research Startup Fund of Zunyi Medical University (BS2021-03), Technology Plan Project of Guizhou Province [Qiankehe Support (2020) No. 4Y179], and Science and Technology Fund Project of Guizhou Provincial Health Commission (gzwkj2021-375).

## Conflict of Interest

The authors declare that the research was conducted in the absence of any commercial or financial relationships that could be construed as a potential conflict of interest.

## Publisher’s Note

All claims expressed in this article are solely those of the authors and do not necessarily represent those of their affiliated organizations, or those of the publisher, the editors and the reviewers. Any product that may be evaluated in this article, or claim that may be made by its manufacturer, is not guaranteed or endorsed by the publisher.
