# Supplementary material for: A Magnetic Resonance Imaging-Based Radiomic Model for the Noninvasive Preoperative Differentiation Between Transitional and Atypical Meningiomas
Source: Front Oncol. 2022 Jan 21;12:811767. doi: 10.3389/fonc.2022.811767 (PMC8815760; doi:10.3389/fonc.2022.811767)
Supplement: Supplementary file 1 [file DataSheet_1.docx]

Supplementary Material

# Supplementary Data

**Image Segmentation**

To evaluate the robustness and reproducibility of the feature extraction, 50 patients of the training cohort were randomly selected after 2 weeks, and their images were segmented again by the same two radiologists to build the re-segmentation set, and 50 patients were randomly selected from each MR scanner to build two MR scanners set for calculating the intra-/interclass correlation coefficients (ICCs).

**Discrimination**

Receiver Operating Curve (ROC) was plotted to show the performance of our models in discriminating TM and AM. Discrimination performance of the models was quantified with the area under the ROC (AUC) value in the training cohort and validated in the independent validation cohort.

**Calibration**

Calibration, which measures the model’s ability to generate predictions that are on average close to the average observed outcome, was plotted to explore the distinguished accuracy in the training cohort and validation cohort.

The Hosmer-Lemeshow (H-L) test is a statistical test for goodness of fit for logistic regression model. It examines how well the percentage of observed TM and AM matches the percentage of predicted TM and AM over deciles of predicted risk. Given our fitted model, using the calculated test statistic, the *P*-value can be calculated as the right-handed tail probability of the corresponding chi-squared distribution. The *P*-values of more than 0.05 were considered well-calibrated and small *P*-value indicates poor fit.

**Decision curve analysis (DCA)**

DCA was conducted to determine the clinical usefulness of the prediction models by deriving the net benefits at different threshold probabilities in the validation cohort, which was yield by plotting net benefit against threshold probability(1).

**Supplementary Results**

The calculation formula for the radiomic nomogram:

Radiomic nomogram score =-2.4461+1.353 × Sex - 0.3482 × Tumour shape + 0.722 × Brain invasion + 4.321 × Fusion radiomic signature. Fusion radiomic signature score=-0.358+0.736 × T1C_WaveletGLSZMwavelet.HHL_Graylevel NonUniformity +0.564 × T1C_SquareRootGLSZM_squareroot_zoneEntropy + 0.665 × T2_ WaveletGLCMwavelet.LLL_JointEnergy + 1.00 × T2_SquareRootGLDM_squareroot_Dependence Entropy.

**Supplementary references**

1. Pencina MJ, D'Agostino RB, Sr., D'Agostino RB, Jr., Vasan RS. Evaluating the added predictive ability of a new marker: from area under the ROC curve to reclassification and beyond. *Stat Med* (2008) 27(2):157-72; discussion 207-12. doi: 10.1002/sim.2929. PubMed PMID: 17569110.

# Supplementary Figures and Tables

## Supplementary Figures


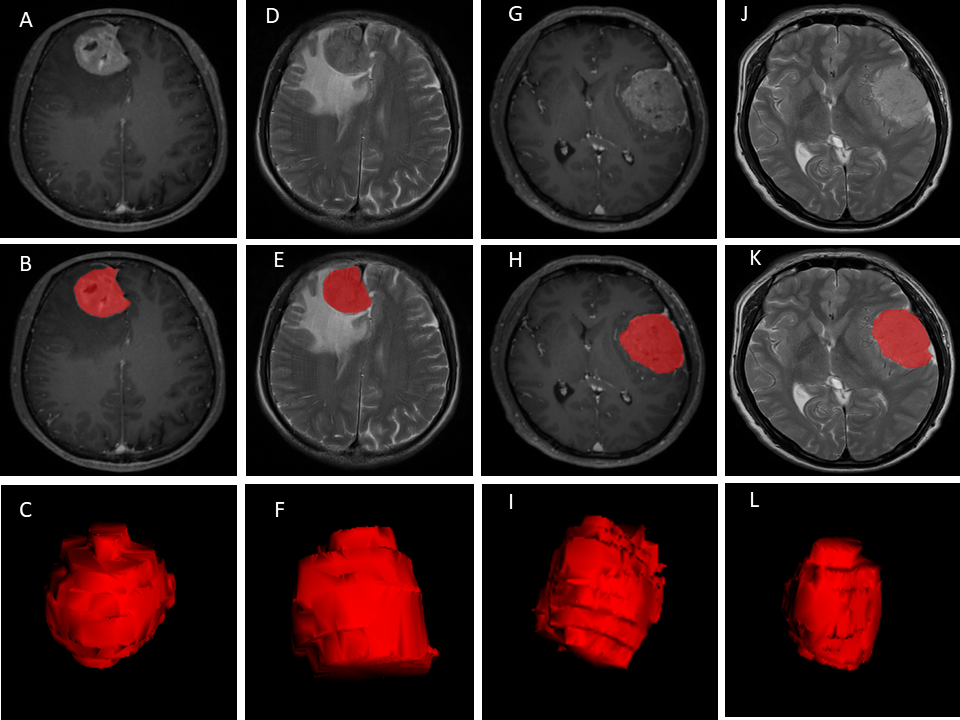


**Supplementary Figure 1.** Axial T1-weighted post-contrast (T1C) and T2-weighted (T2) images of transitional meningioma (TM; **a**, **d**) and atypical meningioma (AM; **g**, **j**), as well as the corresponding manually selected regions of interest (ROIs) (**b**, **e**, **h**, **k**).

.
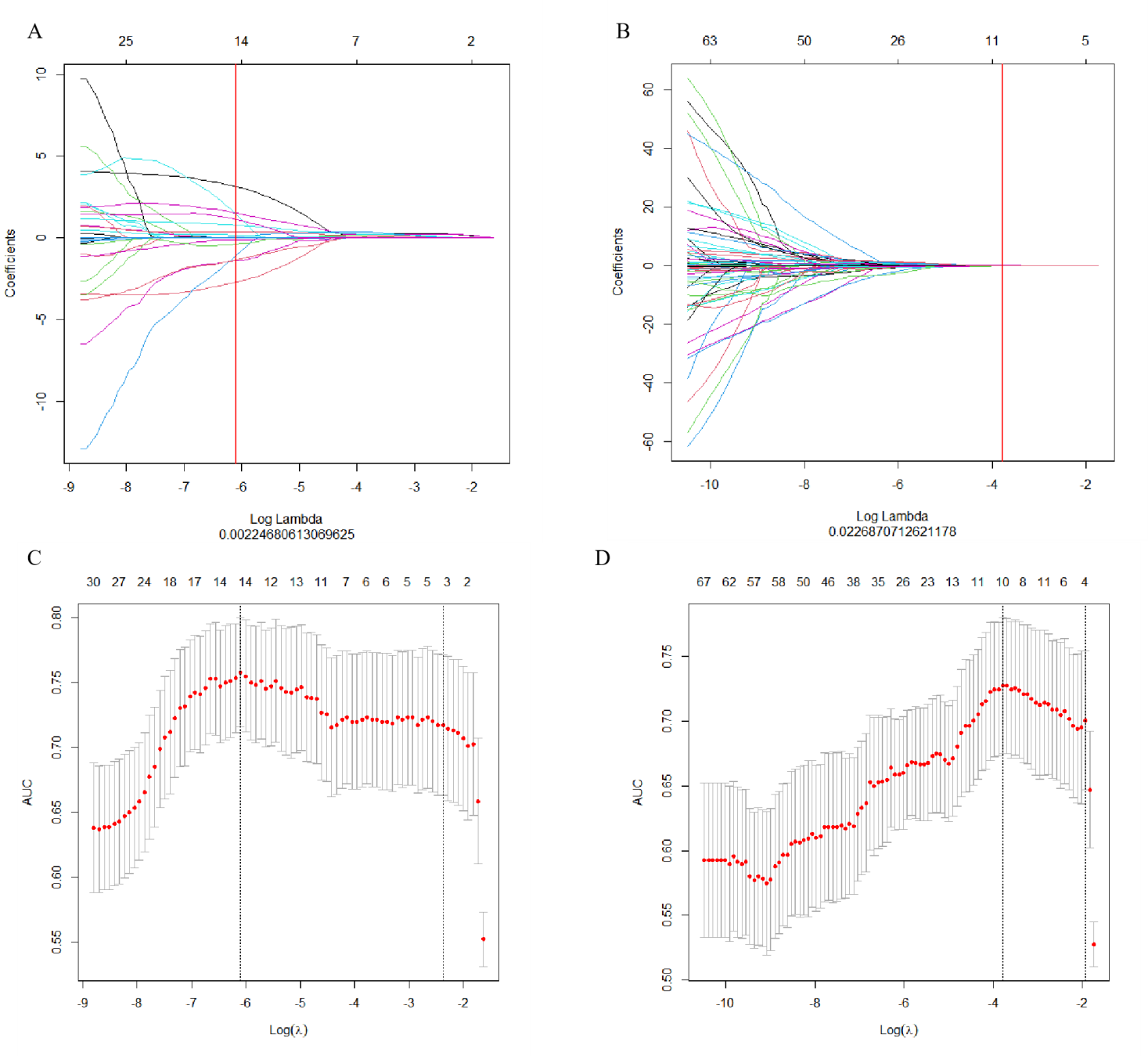
Supplementary Figure 2. Parameters selection by the LASSO regression procedure. A 5-fold cross-validation was used to T1C (C) and T2WI (D) features in the LASSO regression procedure, respectively. AUC was delineated versus log (lambda). Dotted vertical lines were drawn at the optimal values by utilizing the maximum criteria (left dotted line) and the 1-standard error criterion (right dotted line). LASSO, least absolute shrinkage and selection operator.


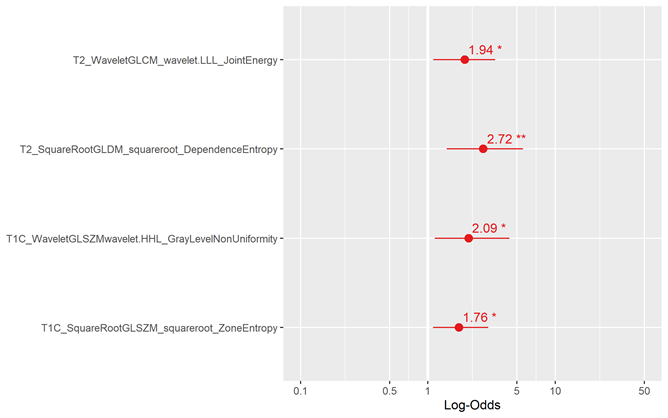


**Supplementary Figure 3**. Odds ratio of the relational radiomic features. The value of odds ratio represents the possibility of diagnosing atypical meningioma (OR ＞ 0), and the larger value indicates that it is more possible to diagnose atypical meningioma.

## Supplementary Tables

**Supplementary Table 1.** MRI parameters of the scanning sequences.

| **Scanner** | **Sequence** | **TR**  **(ms)** | **TE**  **(ms)** | **Matrix** | **Slice Thickness**  **(mm)** | **Slice Gap**  **(mm)** | **Slices** | **Flip**  **Angle** | **Acquisition Time** |
| --- | --- | --- | --- | --- | --- | --- | --- | --- | --- |
| Siemens Verio | T1C  T2 | 1900  3500 | 2.9  91 | 286x256  336x448 | 5.0  5.5 | 5.0  7.7 | 20  18 | 150  150 | 1min8sec  59sec |
| Philips Achieva | T1C  T2 | 7.5  3005 | 3.5  80.0 | 256x256  512x512 | 5.0  6.0 | 5.5  7.0 | 24  18 | 69  100 | 1min2sec  1min21sec |

Abbreviations: T1C, contrast-enhanced T1-weighted imaging; T2, T2-weight imaging; TR, repetition time; TE, echo time.

**Supplementary Table 2.** The weights of each selected radiomics features

| **Radiomics features** | **Coefficients** | ***P* value** |
| --- | --- | --- |
| (intercept) | -0.3584 | 0.0552 |
| T1C_SquareRootGLSZM_squareroot_ZoneEntropy | 0.7355 | 0.0368 |
| T1C_WaveletGLSZMwavelet.HHL_GrayLevelNonUniformity | 0.5636 | 0.0259 |
| T2_WaveletGLCM_wavelet.LLL_JointEnergy | 0.6651 | 0.0130 |
| T2_SquareRootGLDM_squareroot_DependenceEntropy | 1.0009 | 0.0042 |

Note: *P* value less than 0.05 indicates statistical significance.

**Supplementary Table 3.** The *P* value of the correlation between radiomic and clinicoradiological features

| **Radiomic features** | **Training cohort (*P* value)** | | | **Validation cohort (*P* value)** | | |
| --- | --- | --- | --- | --- | --- | --- |
|  | **Sex** | **Tumour shape** | **Brain invasion** | **Sex** | **Tumour shape** | **Brain invasion** |
| T1C_SquareRootGLSZM_squareroot_ZoneEntropy | 0.53 | ＜0.001 | ＜0.001 | 0.14 | 0.011 | 0.002 |
| T1C_WaveletGLSZMwavelet.HHL_GrayLevelNonUniformity | 0.0081 | ＜0.001 | 0.002 | ＜0.001 | ＜0.001 | 0.12 |
| T2_WaveletGLCM_wavelet.LLL_JointEnergy | 0.039 | 0.003 | 0.18 | 0.47 | 0.14 | 0.052 |
| T2_SquareRootGLDM_squareroot_DependenceEntropy | 0.011 | ＜0.001 | 0.022 | 0.18 | 0.024 | 0.057 |

Note: Pearson correlation analysis was used to examine the correlation between the selected radiomic features and clinicoradiological features. *P* value less than 0.05 indicates statistical significance.
